# Dysentery, Complicated with Gastritis

**Published:** 1852-06

**Authors:** C. W. Prather

**Affiliations:** Venice, Butler County, Ohio


*Dysentery, complicated with Gastritis.* By C. W. Prather, M. D., of Venice, Butler County, Ohio.—Dysentery having prevailed in this region as an epidemic, less or more, during the summer-months of the years 1849, '50, and '51, I am induced to prepare a brief account of it for the Lancet.

The first two seasons, it presented itself in such a character, that I was induced to believe that its treatment was based upon false pathological views, from finding that a few small doses of epsom salts and opium would control the disease. But I had not then seen it in its complicated and malignant form, which was left for me to witness, during the summer months of 1851, and which was then of a most aggravated character.

In a few of the cases during this year, the nervous system yielded to the violence of the first shock, was unable to react under the continued violence of the disease, and death terminated the scene in a few days.

The impressions made upon the nervous system, are more vivid in infants than adults, which may at first only produce functional disorders, and if not relieved soon, will terminate in exhausted excitability, or fatal organic disease.

Not having an opportunity of making a post-mortem examination, I was forced to conclude, that the disease in the beginning, was congestion of the colon and rectum, which undoubtedly in a few days terminated in inflammation; but it appeared impossible to tell, at what precise time this took place. In some three or four of the fatal cases, I think inflammation never took place, but the patients died purely from the nervous shock, as early as thirty-six hours from the commencement of the attack.

The different ages, sex, parentage of, mortality, &c., may be seen by the annexed table.

| | Number of cases. | | Number of deaths. | |
|---|---|---|---|---|
| | MALES. | FEMALES. | MALES. | FEMALES. |
| Under 2 years of age | 8 | 11 | 3 | 4 |
| Between 2 and 10 | 19 | 15 | 1 | 5 |
| " 10 " 20 | 11 | 9 | | |
| " 20 " 30 | 14 | 13 | 1 | |
| " 30 " 40 | 3 | 5 | | 1 |
| " 40 " 50 | 3 | 5 | | 1 |
| Above 50 | | 1 | | |
| | — | — | — | — |
| | 58 | 59–117 | 5 | 11–16 |

These statistics are taken from my case book, and comprehend the aggravated cases only; there being at least half that number of mild ones not considered in the above.

The different cases required various modifications of treatment, but in all, opium was the sheet anchor. Calomel, in a few cases proved beneficial, but in the great mass of them, it was more or less an irritant, especially if gastritis was a complication. In a number of cases it was carried to ptyalism, without having any controlling effect upon the disease, which continued on for a number of days after.

Turpentine, in a few cases acted admirably, but in the most of them, it either produced vomiting, or dried up the tongue, and aggravated all of the symptoms.

Creasote certainly produced less benefit in those cases than any other medicine, except in chronic cases, the result of a few of the acute, and also in some after the dysentery had subsided, leaving a slight gastritis which would prevent the patient from convalescing speedily; in such cases it seemed to act with a most happy effect.

Nitrate of silver undoubtedly is perfectly useless. I could see no good effect from it whatever, having used it in different forms, and in every stage of the disease; the effect ascribed to it must undoubtedly be owing to the opium combined with it. Epsom salts in small doses (from 12 grains to a drachm, according to circumstances,) is certainly one of the best adjuvants, but it will not answer if gastritis be present, which is not uncommon in the very beginning, and very frequently makes its appearance during the different stages of the disease.

Astringents are generally of no use in the disease, but if the bowels remain relaxed, with some pain, and occasionally blood and mucus, the sub-nitrate of bismuth will control it frequently.

Opium in all stages of the disease is indispensable; a very good prescription is a combination of calc. magnesia 3 grs., acetate morphia $\frac{1}{8}$ gr., ipecac. $\frac{1}{4}$ gr.; as the opiate allays the disease, the magnesia by accumulation becomes a very gentle aperient.

Fomentation and counter irritants, are very useful adjuvants in the treatment. In one case of six years of age, morphine and fomentations was all that was used; the disease yielded in eight days, and convalesced speedily. When the disease ran a sufficient length of time for the bowels to become ulcerated, the disease always proved fatal; and in those cases a typhoid type of fever would attend, and the ordinary remedies used for it had no effect. It was not of a malarious character, as far as I could judge, or if it was, quinine had no controlling effect.

*Western Lancet.*

